# Antigenic characteristics and genomic analysis of novel EV-A90 enteroviruses isolated in Xinjiang, China

**DOI:** 10.1038/s41598-018-28469-9

**Published:** 2018-07-06

**Authors:** Keqiang Huang, Yong Zhang, Yang Song, Hui Cui, Dongmei Yan, Shuangli Zhu, Qiang Sun, Haishu Tang, Dongyan Wang, Wenbo Xu

**Affiliations:** 1 WHO WPRO Regional Polio Reference Laboratory and Ministry of Health Key Laboratory for Medical Virology, National Institute for Viral Disease Control and Prevention, Chinese Center for Disease Control and Prevention, Beijing, People’s Republic of China; 2 Xinjiang Uygur Autonomous Region Center for Disease Control and Prevention, Urumqi City, Xinjiang Uygur Autonomous Region People’s Republic of China; 3 Anhui University of Science and Technology, Huainan city, Anhui province People’s Republic of China

## Abstract

Enterovirus A90 (EV-A90) is a novel serotype of enterovirus A species that is rarely reported. Here, we isolated five enteroviruses from patients with acute flaccid paralysis in Hotan and Kashgar cities in Xinjiang, China that were identified as EV-A90 by molecular typing. The *VP1* sequences of these Xinjiang EV-A90 strains showed 88.4–89% nucleotide sequence identity to the prototype EV-A90 strain; however, genome analysis indicated complex recombination events in *P2* and *P*3 regions. Next, the seroprevalence of EV-A90 was examined in 49 serum specimens collected in Hotan and Kashgar, and 37.5% were EV-A90 antibody positive (>1:8), with a geometric mean titre (GMT) of 1:10.47. The low positive rate and GMT suggest a low-level EV-A90 epidemic in Xinjiang. Two of the five Xinjiang EV-A90 strains were temperature sensitive, and three were temperature resistant, and a comparative genomics analysis suggested that an amino acid substitution (H1799Y) in the 3D^pol^ region was related to temperature sensitivity. Although the epidemic strength is low, some EV-A90 strains were temperature resistant, which is suggestive of strong virulence and transmission capacity. This study expanded the number of EV-A90 in GenBank and provided basic data that may be useful for studying the molecular epidemiology of EV-A90.

## Introduction

Enteroviruses (EVs) are members of genus *Enterovirus* in the family *Picornaviridae* and the order *Picornavirales*. The *Enterovirus* genus is comprised of enteroviruses and rhinoviruses, and species EV-A to EV-D and RV-A to RV-C, respectively, are the main causative agents of human diseases^1^. In the past, the neutralization assay was used as the gold standard for enterovirus serotype identification. However, this method has some disadvantages, as it is time consuming, labour intensive, and subject to antisera shortages, etc. In addition, antigenic variants and cross-reactivity can confound the results. With the development of molecular methods over the past two decades, the neutralization assay has been gradually replaced by molecular typing methods, which overcome some of the shortcomings of the neutralization assay and provide identification information for novel enteroviruses^2,3^. In addition, numerous novel enteroviruses have been identified and classified with these molecular typing techniques. Currently, there are more than 110 identified EV types.

EVs possess a single-stranded, positive-sense RNA genome of approximately 7400–7500 nucleotides that is comprised of a single open reading frame (ORF) flanked by two non-translated regions (NTRs) at the 5′ and 3′ ends^4^. The polyprotein translated from the positive-strand RNA is cleaved into a structural polyprotein (P1) and other non-structural polyproteins. And the structural protein is further cleaved to yield four viral proteins (VP1–4). Most neutralization epitopes are located in the *VP1* protein, and there are variations in the amino acid composition both within and between species, which are important for EV classification^5^. EV serotypes can be further divided into different genotypes or sub-genotypes based on the *VP1* region sequences.

Enterovirus type EV-A90 is a novel enterovirus that has been associated with acute flaccid paralysis (AFP). It was firstly identified and classified in 2005 with a molecular typing method, and the first EV-A90 strain, BAN99-10399, was assigned as the prototype strain^6^. Currently, there are six full-length EV-A90 genome sequences and several entire *VP1* sequences in GenBank^6–10^. In mainland of China, EV-A90 has been rarely reported, and the only previous reports were in Shandong province, where EV-A90 was isolated from three children in 2001 and 2003^11^.

In this study, we determined the full-length genome sequences of five EV-A90 strains (HT-MYH12F/XJ/CHN/2011, HT-HTH08F/XJ/CHN/2011, HT-PSH40F/XJ/CHN/2011, KS-KSH13F/XJ/CHN/2011, and KS-SCH05F/XJ/CHN/2011, hereafter referred to as HT-MYH12F, HT-HTH08F, HT-PSH40F, KS-KSH13F, and KS-SCH05F, respectively) that were isolated in southern of Xinjiang Uygur Autonomous Region of China.

## Results

### Virus harvest and viral titre determination

Stool specimens were inoculated into rhabdomyosarcoma (RD) cells. When a complete cytopathic effect (CPE) was observed during a five-day observation period, viruses were harvested^12^. After a fixed gradient dilution and re-inoculation into a 96-well plate, the viral titre was calculated according to the Behrens-Kärber formula. All of the samples were determined to have a cell culture infective dose of 10^5^–10^6^ in 0.05 mL of cell culture (CCID_50_/0.05 mL).

### Molecular typing and sequence analysis

Using molecular typing methods, we identified all five isolates in this study as EV-A90. The VP1 regions of these isolates showed 88.4–89.0% nucleotide sequence similarity and 98.2–98.9% amino acid sequence similarity to the prototype strain. The genomes of these five EV-A90 strains were 7,423–7,425 nucleotides in length, with a 5′-NTR of 747–749 nucleotides, a 94-nucleotide 3′-NTR preceding the poly (A) structure, and a single ORF of 6,582 nucleotides encoding 2,194 amino acids.

In the comparative analysis, 81 enterovirus prototype strains were used as the reference sequences, including 18 EV-A strains, 55 EV-B strains, 7 EV-C strains, and 1 EV-D strain. As shown in Table 1, the Xinjiang EV-A90 isolates were highly similar to each other and to some of the other EV-A in some regions. For example, they showed 82.6% nucleotide (100% amino acid) identity to EV-A91 (AY697461) in the *VP4* region, 88.2–89.2% nucleotide (97.9–99.3% amino acid) identity in the *2B* and *2 C* regions, and 87.2–92.1% nucleotide (96.4–100% amino acid) identity in the *P3* region. They also showed >85% nucleotide (90% amino acid) identity to EV-A121 (KU355877), EV-A76 (AY607458), EV-A89 (AY697459), and EV-A92 (EF667344) in the *2B* region or other non-structural regions. This suggested that complex recombination events probably occurred in the *P2* and *P3* regions of the Xinjiang EV-A90 strains.

**Table 1:** Comparison among five EV-A90 strains from Xinjiang Uygur Autonomous region of China, the EV-A90 prototype strain (BAN99-10399) and other Enterovirus prototype strains in nucleotide sequence and deduced amino acid sequence identities.

Region	%nucleotide identity(%amino acid identity)					
	Comparison with BAN99-10399		Comparison with other EV-A		Comparison with other EV	
	Nucleotide	Amino acid	Nucleotide	Amino acid	Nucleotide	Amino acid
5′-NTR	92.1–92.9	—	26.8–42.6	—	57.1–82.4	—
VP4	87.9–89.8	98.5	62.8–82.6	63.7–100	53.6–70.5	53.6–78.2
VP2	88.6–88.8	98.4–99.2	64.1–72.1	72.6–81.1	50.1–57.0	50.0–58.8
VP3	89.7–90.1	98.7–99.5	63.7–72.4	70.9–83.5	47.8–54.7	42.8–53.2
*VP1*	88.4–89.0	98.2–98.9	53.1–66.5	56.0–73.6	35.2–48.4	29.2–36.6
2A	87.3–88.0	94.6–95.3	64.0–69.7	68.6–77.3	56.8–71.7	5.9–75.8
2B	82.4–83.8	97.9–98.9	66.3–89.8	75.7–100	49.8–61.9	42.4–60.2
2C	90.0–90.5	99.0–99.6	73.5–90.8	83.2–99.3	58.6–64.1	59.5–66.3
3A	88.6–89.8	96.4–97.6	66.2–92.1	68.2–97.6	49.4–60.6	48.3–56.1
3B	83.3–86.3	100.0	54.5–93.9	61.9–100	43.9–60.6	39.1–59.0
3C	87.7–89.2	98.3–98.9	69.2–89.2	82.5–97.8	54.8–60.8	53.5–59.0
3D	90.6–91.1	98.2–98.4	71.7–92.2	82.2–98.9	61.3–65.5	64.7–69.9
3′-NTR	92.5–94.6	—	13.8–94.6	—	12.6–52.3	—

### Phylogenetic analysis of global EV-A90 strains and evolutionary distance estimation

**Figure 1:**
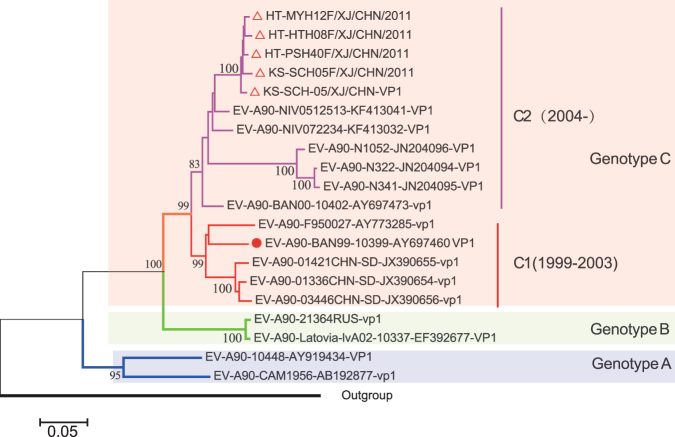
Phylogenetic tree of global EV-A90 based on entire VP1 sequence. Isolates from Xinjiang were marked with  and the prototype of EV-A90 (BAN00-10399) was marked with . The phylogenetic tree was reconstructed using neighbor-joining method with the substitution model of maximum composite likelihood model.

We collected all the available *VP1* sequences and full-length genome sequences of EV-A90 strains from GenBank. Then, we constructed phylogenetic trees based on the sequences of the *VP1* region (Fig. 1) after sequence screening, multiple alignment, and shape optimization. Bootstrapping values greater than 80% were considered statistically significant^13^. According to the phylogenetic tree based on the *VP1* region, the EV-A90 strains are divided into 3 clusters (A–C), and the strains in cluster C are further divided into 2 sub-clusters (C1 and C2) based on nucleotide divergence. Strains belong to C genotype become major prevalent cluster, which is different from the early genotypes that have only a few sequences published. We inferred that viruses in Cluster C are possibly more actively varying, especially those in the C2 sub-cluster. As shown in Table 2, the p-distance among Clusters A, B, and C were 0.2632 ± 0.0172, 0.2609 ± 0.0168, and 0.1797 ± 0.0134 (95% confidence interval [CI]), respectively, and the maximum nucleotide differences between two Clusters were 22.9%, 23.7%, and 16.4%. Moreover, the p-distance within Cluster C was 0.1138 ± 0.0088 (95% CI), and the maximum difference between sub-clusters C1 and C2 was 12.0%. According to the strategies for the classification of EV-A71 genotypes^14^, clusters A, B, and C were three different genotypes (Genotype A, B and C), and genotype C was further divided into two sub-genotypes (C1 and C2).

**Table 2:** The nucleotide (deduced amino acid) similarity and the P-distance between groups (sub-groups) were calculated. The similarity data was shown in the first two parts. Another is the P-distance between groups (subgroups) in the 95% confidence interval. And the standard error was shown below each data.

Cluster		Nucleotide Similarity %			Amino acid similarity%			P-distance in the 95%confidence interval		
		A	B	C	A	B	C	A	B	C
				C1			C1			C1
A										
B		77.1–78.8			91.1–92.8			0.2460–0.2804 SE:0.0172		
C	C1	76.3–78.3	83.6–85.7		90.5–92.8	94.2–97.2		0.2475–0.2743 SE:0.0168	0.1663–0.1931 SE:0.0134	
	C2	77.6–78.9	83.8–86.1	88.0–94.9	91.5–93.2	95.2–96.9	94.5–99.3			0.1050–0.1226 SE:0.0088

### Recombination analysis of the Xinjiang EV-A90 strains

**Figure 2:**
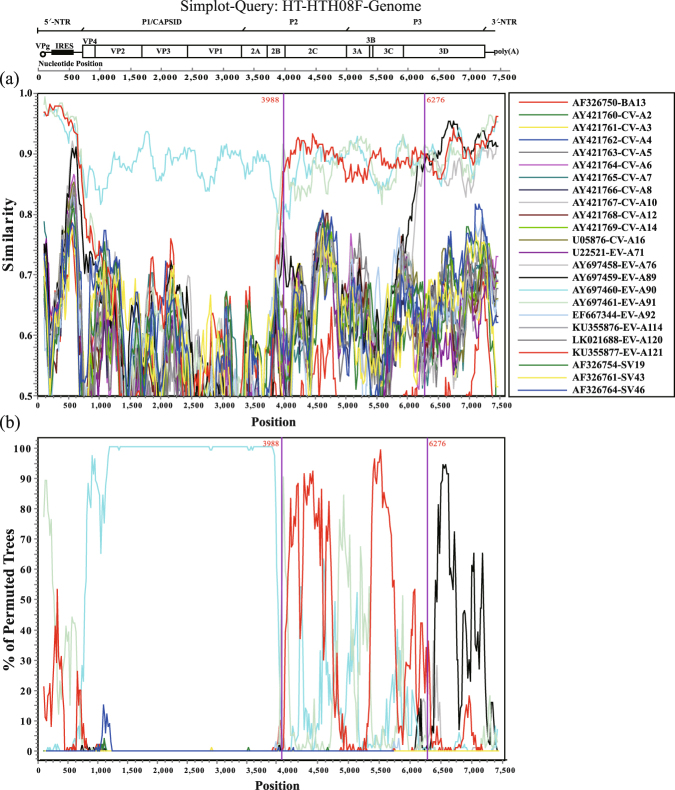
Potential recombination analysis of the whole genome of the Xinjiang EV-A90 strains. Similarity analysis (**a**) and bootscaning analysis (**b**) were performed in a 200-nt sliding window. Each point indicated similarity between the Xinjiang strain and other EV-A strains in a 20-nt moving step. Kimura (2-parameter) model was used in the analysis.

**Figure 3:**
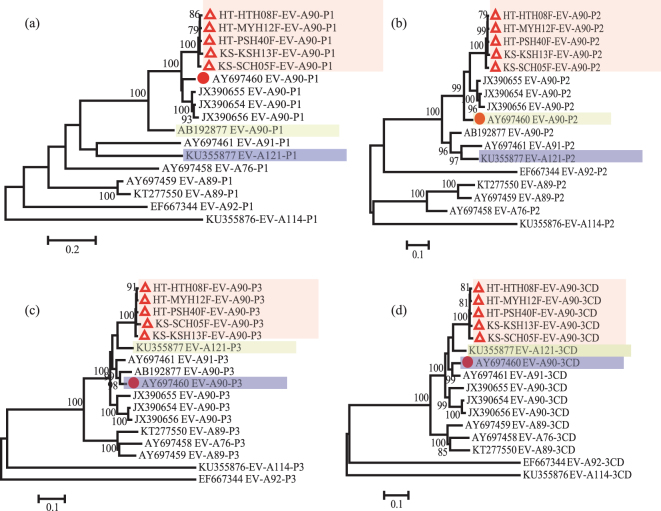
Phylogenetic tree of genetic fragments for EV-A90 and other similar serotypes, including P1, P2, P3 and 3CD, were illustrated in figure (**a**–**d**). Isolates in this research were marked with  and the prototype of EV-A90 (BAN00-10399) was marked with  . The phylogenetic tree was reconstructed using maximum likelihood method, in which GTR + I + G model was used in P1 fragment and TN93 + I + G model was used in the rest fragment. We also put forward a hypothesis between the major parental type (yellow) and the minor parental type (blue) because of recombination.

We used Simplot (V3.5.1) to analyse the five Xinjiang EV-A90 strains for potential recombination, and the results were quite similar, so one EV-A90 sequence (HT-HTH08F) was selected as a reference strain. The similarity plot and genome bootscanning analysis of HT-HTH08F were shown in Fig. 2 indicated that the *P1* regions of the five strains were homologous to that of the prototype EV-A90 strain, whereas the *P2* and *P3* regions, specifically from the second half of the *2B* region to the 3′-NTR, were more similar to EV-A121 and EV-A91. In addition, the sequence from *3D* to the 3′-NTR was also fairly similarity to EV-A89 and EV-A76. This suggested that recombination events occurred in the Xinjiang EV-A90 strains, likely with some other EV-A types, between *2B* and *3D*. By verifying of maximum likelihood (ML) tree (Fig. 3), we found an obvious topological exchange between EV-A90 and EV-A121 from *P3* region (or *3CD* region). It possibly proved a recombination occurred. We hardly found the same phenomenon in *P2* region (or after *2B* region) even though the EV-A90 strains also partially lost their original genetic characteristics in *P2* region.

### Neutralization assay and seroprevalence evaluation

In our study, 49 serum samples were collected from healthy children in Xinjiang who were ≤5 years old. Of these samples, 25 were collected from Hotan city, and 24 were collected from Kashgar city. In the neutralization assay in 96-well plates, only 18 samples (>1:8) were positive for EV-A90, for a total positive rate of 36.73%. The geometric mean titre (GMT) was 1:10.47 for all positive samples. The EV-A90 neutralization antibody titres are shown in Table 3. The results of the neutralization assay showed an obvious difference between the two cities (Z = 3.2344, P < 0.01). In Hotan, the GMT was 1:9.19, while in Kashgar, it was 1:20.16. However, in general, the seroprevalence of EV-A90 in both cities was lower than that of other enterovirus serotypes, such as EV-A71^15^.

**Table 3:** The compositions of EV-A90 neutralization antibody titers.

Titers	Kashgar city		Hotan city		Total(%)
	Sample amounts	Ratio (%)	Sample amounts	Ratio (%)	
<1:8	9	37.5%	22	88%	31 (63.3%)
1:8–1:64	15	62.5%	3	12%	18 (36.7%)
>1:64	0	0	0	0	0
Total	24	100%	25	100%	49 (100%)

### Xinjiang EV-A90 strains showed different temperature-related phenotypes

**Figure 4:**
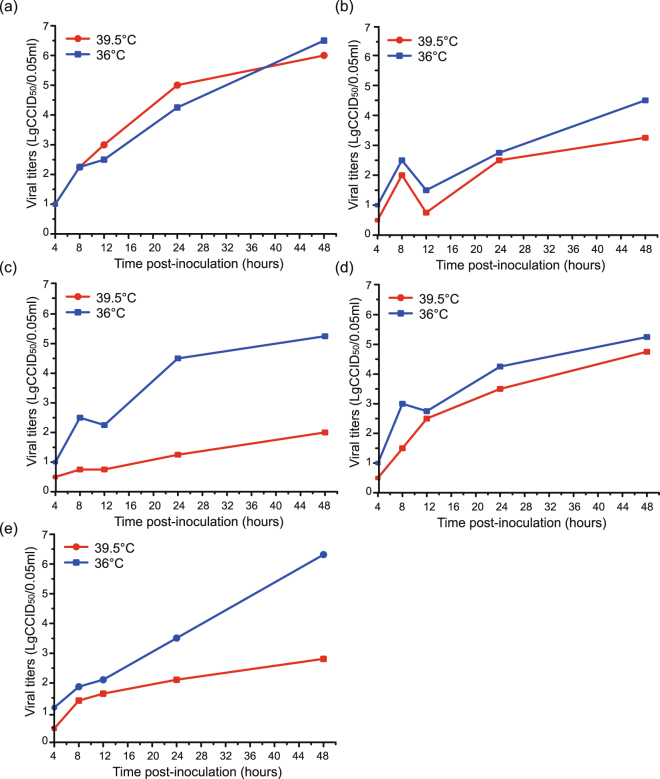
The viral titers of five EV-A90 strains in different time were drawn in the coordinate system. The blue line represented the growth curve in 36 °C and the red one represented the growth curve in 39.5 °C.

Temperature sensitivity is an important characteristic of enteroviruses. It usually serves as an *in vitro* marker for the attenuation of poliovirus vaccine strains and EV-A71, although the link between temperature sensitivity and attenuation may not be straight forward, it could serve as an indicator of virulence in Enterovirus^16,17^. The Poliovirus Sabin strain is a good example for understanding the importance of temperature sensitivity. Based on our observations, three strains (HT-MYH12F, HT-HTH08F, and KS-KSH13F) were temperature resistant and two others (HT-PSH40F and KS-SCH05F) were temperature sensitive (Fig. 4). After a 48 h incubation, the maximum viral titre of KS-SCH05F and HT-PSH40F at 36 °C was >10^5^ TCID_50_, whereas their titres at 39.5 °C were ≤10^3^ TCID_50_. In contrast, the titre of the other three strains did not differ significantly when they were incubated at these two temperatures. Generally speaking, we considered a strain to be temperature sensitive when the logarithm of the viral titre of the strain at 36 °C was twice the viral titre of the strain at 39.5 °C. The results showed that HT-MYH12F, HT-HTH08F, and KS-KSH13F replicated to a greater degree at the higher temperature (39.5 °C).

To investigate the possible mechanism underlying the difference in temperature sensitivity among these strains, they were screened for nucleotide and amino acid substitutions. A total of 26 nucleotide substitutions in the NTRs and 29 amino acid substitutions in the ORF were detected and are summarized in Table 4. The substitutions were randomly distributed throughout the genome; however, one amino acid substitution, a histidine to tyrosine in 3Dpol was interesting. In the two temperature-sensitive strains, the amino acid at this locus is a histidine, whereas in the three temperature-resistant strains, this amino acid is a tyrosine. We also compared the base composition and codon bias between temperature-sensitive and temperature-resistant strains; however, little information was obtained. The strains in both groups showed 44.6% G + C content, and the amino acid total compositions of the five strains were identical.

**Table 4:** 26 nucleotide substitutions in NTR and 29 amino acid substitutions in ORF among five EV-A90 strains. The position of nucleotide was calculated from the first nucleotide of its NTR after a multi-alignment by ClustalW. But there was no observation in *VP4* and *3B*.

**Region & Position (nucleotide)**									**Strain**	**Region &Position (amino acid)**									
5′-*NTR*										*VP2*			*VP3*				*VP1*		
18	19	32	64	117	119	122	149	171		142	195	196	407	459	470	548	585	669	708
T	—	A	C	T	T	—	A	**A**	**HT-HTH08F**	**N**	**A**	**I**	**T**	**V**	**T**	**K**	**N**	**H**	**T**
G	—	.	.	.	.	—	.	**G**	**HT-MYH12F**	**S**	.	.	**S**	**I**	**A**	.	.	**N**	**A**
.	—	G	T	C	.	—	.	**G**	**KS-KSH13F**	**S**	**V**	**V**	.	**I**	**A**	.	**D**	**N**	**A**
G	T	.	.	.	.	T	.	**G**	**HT-PSH40F**	**S**	.	.	.	**I**	**A**	**G**	.	**N**	**A**
G	—	.	.	.	A	—	G	**G**	**KS-SCH05F**	**S**	.	.	.	**I**	**A**	.	.	**N**	**A**
5′-*NTR*									**Strain**	*VP1*		*2A*			*2B*	*2C*			*3A*
234	260	265	338	538	645	683	691	701		711	855	865	925	967	1044	1141	1166	1283	1452
T	T	T	G	C	—	A	A	**A**	**HT-HTH08F**	**A**	**V**	**V**	**V**	**A**	**F**	**I**	**P**	**V**	**S**
.	.	.	.	.	—	.	.	.	**HT-MYH12F**	**T**	**A**	.	.	**T**	.	.	.	.	**T**
.	.	.	.	.	—	.	G	.	**KS-KSH13F**	.	.	.	.	**T**	.	.	**A**	.	**T**
C	.	.	A	.	—	G	.	.	**HT-PSH40F**	.	.	**M**	.	**T**	.	.	.	.	**T**
.	C	C	.	T	A	.	G	**G**	**KS-SCH05F**	.	.	.	**I**	**T**	**Y**	**M**	.	**A**	**T**
5′-*NTR*				3′-*NTR*					**Strain**	*3A*	*3C*		*3D*						
712	736	739	746	7334	7357	7385	7423			1484	1614	1667	1799	1961	1998	2158	2166	2185	
**T**	**C**	**T**	**A**	**T**	**T**	**C**	**T**		**HT-HTH08F**	**I**	**Q**	**V**	Y	**F**	**E**	**R**	**D**	**L**	
.	.	.	.	.	**C**	.	.		**HT-MYH12F**	.	.	**I**	Y	.	.	.	**N**	.	
**C**	.	.	**G**	.	**C**	**T**	.		**KS-KSH13F**	.	.	.	Y	.	.	**K**	.	.	
.	.	.	.	.	**C**	.	**G**		**HT-PSH40F**	**V**	.	.	H	.	.	.	.	**M**	
.	**T**	**C**	.	**C**	**C**	.	.		**KS-SCH05F**	.	**H**	.	H	**L**	**D**	.	.	.	

## Discussion

The novel enterovirus serotype is a classification that first appeared after the traditional serotyping method was replaced by molecular typing methods^18^. Isolates with >75% nucleotide similarity (or >85% amino acid similarity) in the *VP1* region are considered to be of the same serotype^5,19^. If this condition is not met, then the strains may belong to different serotypes. Based on molecular typing, many novel enterovirus serotypes have been defined and re-classified.

In mainland of China, many novel enterovirus serotypes were identified through the AFP case surveillance system. We reviewed the novel enterovirus serotypes identified in China in recent years, including EV-A76^20^, EV-A89^21^, EV-B81^13^, EV-B111^22^, and EV-C96^23^, and found that a sensitive surveillance system plays an very important role in finding novel enteroviruses, even when the surveillance system is not specific for enteroviruses.

The gold standard to distinguish enteroviruses serotypes is molecular typing method that based on the difference of *VP1* sequences similarity. Although there are no global criteria for the classification of EV genotypes, rather, the ranges of percentage similarities between (intra-typic) clusters and subclusters often overlap, suggesting that percentage similarities cannot always be used for unambiguous genotype/genogroup designations, 15% difference in nucleotide sequences for identifying enterovirus genotypes plays an important role in molecular epidemiology research. Brown and Oberste published an article^14^ that has strong impact on classification of enterovirus genotype and subgenotype. EV-A71, an enterovirus serotype, use this standard for genotyping. Followed that, E-11^24^, CV-A16^25^ as well as CV-A6^26^ has been classified to subgenotype used the same standard. Besides, the genotyping and subgenotyping standards for EV-A71 and CV-A16 have been accepted by RIVM, who developed enteroviruses molecular typing tool^27^. As to the global distribution of EV-A90, previous studies showed that it was divided into two genotypes, and genotype B was the major type, which is spreading in Asia and Europe^11^. However, a Latvian EV-A90 strain^7^ (accession number EF392677) and a Russian EV-A90 strain^10^ (accession number KC879486) clearly form a separate branch and should be considered carefully. Whether these strains are genotype A or B is difficult to determine as strains in this branch have at least 16% nucleotide difference among them. According to the criteria for the differentiation of enterovirus genotypes, EV-A90 strains should to be divided into three genotypes (A, B, and C), with sub-genotypes C1 and C2 in genotype C (previously classified as genotype B). And the C genotype of EV-A90 is presently the major type in circulation. However, the definition and number of genotypes are affected by the numbers of the reported cases or the published sequences, thus, these results may change as viruses evolve and as more sequences are reported.

It is generally accepted that mismatch mutations and recombination are the principal mechanisms driving the evolution of enteroviruses^28,29^. Recombination, especially intertypic recombination, is often observed in the non-structural genomic regions encoding enzymes and small active molecules, which may lead to changes in their environmental adaptations and disease severity^29^. An early study showed high nucleotide sequence similarity among EV-A90, EV-A76, EV-A89, EV-A91, and EV-A121 in the *3D* region^30^. Not only can these studies help to identify recombination events in enteroviruses, but it is also important for screening of possible parental donors. In our study, the similarity and genome bootscanning analysis suggested that all of the five Xinjiang EV-A90 strains were recombinants, especially in *P3* (or *3 CD*) region, we proved them a recombination with EV-A121. In addition, we found that the sequences of the Xinjiang strains in part of the *P2* region was highly similar to to EV-A76, EV-A89, and EV-A91. However, we rarely found the slightly different topological characteristics between *P1* and *P2* regions. Certainly, we can’t found the major parental type in *P2* region, which is possibly affected by some unidentified EV-A types. Therefore, the possibility that Xinjiang EV-A90 recombined with an unidentified EV-A serotype enterovirus cannot be excluded. Detailed surveillance data for novel enteroviruses in different areas over different time periods are needed for further study.

Based on the seroepidemiological results of this study, EV-A90 enteroviruses are present at low levels, and the low average seroprevalence of EV-A90 antibodies suggests that there has not been a large-scale epidemic in Xinjiang. Because the levels of neutralization antibody levels are dynamic, the level within a population is an objective indicator of prevalence and transmission. Even though there are significant differences between Kashgar and Hotan, none of the strains were likely acquired in a large-scale epidemic. Because the strain (KS-SCH05F) used in the neutralization assay was isolated from Kashgar, it was expected that the overall level in Kashgar would be slightly higher than that in Hotan.

It is very interesting that, among the five tested Xinjiang EV-A90 strains, three (HT-MYH12F, HT-HTH08F, and KS-KSH13F) were temperature resistant and two (strain HT-PSH40F and KS-SCH05F) were temperature sensitive. Among the nucleotide and amino acid differences in the five Xinjiang EV-A90 strains, it seemed that an amino acid substitution (histidine to tyrosine) in the 3D^pol^ region may be related to the temperature-sensitive phenotype, because the amino acid in the two temperature-sensitive strains is histidine while that in the three temperature-resistant strains is tyrosine. Interestingly, this amino acid substitution was also observed in a type I attenuated poliovirus vaccine strain (type I Sabin strain)^31^. In previous studies, substitutions in 3C and 3D were often related to changes in biological characteristics, including virulence, temperature-sensitivity, etc.^32^. However, additional evidence is needed to support this hypothesis, and our team is currently using reverse genetics to verify the role of this site.

In conclusion, we reported the full-length genome sequences of five EV-A90 strains isolated during AFP surveillance in the Xinjiang Uygur Autonomous Region of China. This is the first report of EV-A90 in Xinjiang, and at present, the number of EV-A90 strains worldwide is still very limited. Sequence analysis suggested that these five EV-A90 strains have high genetic diversity compared with the prototype strain, suggesting intertypic recombination within the non-structural protein-encoding region of all five strains, and extensive genetic exchange with other EV-A serotypes, such as EV-A89, EV-A76, EV-A91, and EV-A121. According to the seroepidemiology survey, EV-A90 remains a low-level epidemic in Xinjiang. However, these viruses may have strong virulence or transmission capacity, since some of the isolated Xinjiang EV-A90 strains were temperature resistant. Hence, EV-A90 has the potential to become a more common strain. This study expands the number of EV-A90 whole genome sequences in GenBank and provides valuable information regarding the molecular epidemiology of EV-A90.

## Methods

### Sample collection and virus isolation

This study involved no human participants or experimentation. The only materials used in our study were stool samples that were collected during a long-term AFP surveillance program for public health purposes. The AFP surveillance system is run by the national laboratory network in support of the polio eradication strategy. Written informed consent for the use of their clinical samples was obtained from all individuals involved in this study. This study was approved by the Ethics Review Committee of the National Institute for Viral Disease Control and Prevention (NIVDC), Chinese Center for Disease Control and Prevention. All experimental protocols were approved by the NIVDC, and the methods were carried out in accordance with the approved guidelines.

The Xinjiang EV-A90 strains HT-MYH12F, HT-HTH08F, and HT-PSH40F were isolated from stool samples of patients with AFP residing in Hotan city, Xinjiang, China. The other two strains KS-KSH13F and KS-SCH05F were similarly isolated from Kashgar city, Xinjiang, China. We used the RD cell line for virus isolation. Each strain was purified by plague assays and cultivation of serial dilution. The cell line was provided by the WHO Global Poliovirus Specialized Laboratory in the USA and was originally purchased from American Type Culture Collection (Manassas, VA, USA). Infected cell cultures were harvested after complete CPE was observed.

In the seroprevalence study of EV-A90 antibodies, 49 healthy children ≤5 years of age were surveyed. Serum samples were collected from these 49 children in 2013 for seroepidemiological analysis of enteroviruses, with informed parental consent, by the Xinjiang Center for Disease Control and Prevention. The samples included 25 from Hotan and 24 from Kashgar, which are the two cities where the enterovirus strains were isolated. The same serum samples were used previously for EV-A89 and EV-B106 seroepidemiology studies^21,33^. None of the children showed any signs of disease at sample collection.

### Molecular typing and full-length genome sequencing

Viral RNA was extracted from 100 µL of viral isolate with the QIAamp Viral RNA Mini Kit (Qiagen, USA). The primer pair E488 and E486^34^ was used to amplify a portion of the *VP1* region. The PCR products were purified using the QIAquick PCR purification kit (Qiagen, Germany) and sequenced in both directions from each strand using an ABI 3130 Genetic Analyser (Applied Biosystems, Foster City, CA, USA). The obtained partial *VP1* sequences were analysed by comparison to the sequences in GenBank using the BLAST server and were typed using the EV Genotyping Tool.

Amplification of the full-length genome was performed in three steps, in which three overlapping RNA fragments were amplified. Reverse transcription-polymerase chain reaction (RT-PCR) was performed using the SuperScript III One-Step RT-PCR System with Platinum Taq High Fidelity DNA Polymerase (Thermo Fisher Scientific, USA). Then amplicons were purified with the QIAquick PCR purification Kit (Qiagen, Germany) and sequenced using an ABI 3130 Genetic Analyser (Applied Biosystems, Hitachi, Japan) by the dideoxy chain termination method. Each nucleotide was sequenced at least once. Some primer pairs used for PCR and sequencing were designed with Primer 5.0 software, and their parameters were assessed on the OligoCalc website (http://biotools.nubic.northwestern.edu/OligoCalc.html)^35^. The rest of genome was determined with a primer walking strategy. The primer pairs used for each step are listed in Table 5.

**Table 5:** PCR and sequencing primers.

Primer	Position	Primer sequence (5′ to 3′)	Orientation	Reference
0001S48	—	GGGGACAAGTTTGTACAAAAAAGCAGGCTTTAAAACAGCTCTGGGGTT	Forward	^39^
EV-A90-490S	490–509	CCTAACCACGGAGCAAGTAC	Forward	This study
EV-A90-589A	570–589	TTGTCACCATAAGCAGCCAT	Reverse	This study
EV-A90-1784A	1763–1784	CTGAGACACCATCATCTGTAGT	Reverse	This study
EV-A90-2347S	2347–2368	GAGCCCAACATCAGCTTACATC	Forward	This study
EV-A90-2607A	2587–2607	CCAGTCTCCGCTGCTTGCAG	Reverse	This study
EV-A90-3036S	3036–3056	GCAGCAGCGTATCAATGGTTC	Forward	This study
EV-A90-3487S	3487–3508	GTGACACCATAGCACGTTGCTC	Forward	This study
EV-A90-3964A	3945–3964	TCACTCCTAACAACTATCAC	Reverse	This study
EV-A90-4277A	4259–4277	CCTGACTAGCTGCTGATTG	Reverse	This study
EV-A90-4868S	4868–4885	GATGCTGCTAGAGCTGCC	Forward	This study
EV-A90-5015S	5015–5032	CAGAGAATACAACAACCG	Forward	This study
EV-A90-5099A	5080–5099	GCTGGTTTGTCAAGGGTTAT	Reverse	This study
EV-A90-6657S	6657–6679	GCTAGTCTTTCTCCTGCTTGGTT	Forward	This study
EV-A90-6827A	6806–6827	GCTAGTACCAGAGCATCCAGAT	Reverse	This study
7500A		GGGGACCACTTTGTACAAGAAAGCTGGG(T)_24_	Reverse	^39^

### Sequence bioinformatics analysis

The nucleotide (or deduced amino acid) sequences of the Xinjiang EV-A90 strains and other enteroviruses were compared by pairwise alignment using MEGA (version 6.06)^36^. The similarity matrix was processed with BioEdit (version 7.0.9.0)^37^. The neighbour-joining method with the maximum likelihood composite model was used to construct the phylogenetic trees. The bootstrap analysis was performed with 1000 pseudo-replicates. For the potential recombination analysis, we used Simplot 3.5.1^38^. The result is shown in a 200-nt sliding window, in which each point represented similarity between the Xinjiang EV-A90 strain and other EV-A types. Besides, we also constructed ML tree for verifying recombination using suitable substitution model tested by MEGA.

### Neutralization assay against EV-A90

In our study, antibody detection and seroprevalence evaluation were performed by micro-neutralization assay^15^. Before use, each serum sample was inactivated by incubation at 56 °C for 30 min. Then, each sample was serially diluted (1:4 to 1:1024), and aliquots (50 µL) of five different concentrations were placed in duplicate into a 96-well plate. Next, we added virus (50 µL) to each well and confirmed that each well was 100 TCID_50_. After 2 h of neutralization at 36 °C in a CO_2_ incubator, we added the same volume of RD cells to each well. Two wells in each column were used as serum controls. We also used a new plate for a cell control and virus control that we called a virus back-titration. All of the plates were incubated for 7 days. Only when the virus titre in the back-titration plate was between 32 and 320 TCID_50_ and when the cell control was acceptable did we judge the result as effective. Then, the highest dilution of serum where 50% of the cultures were protected from CPE was recorded. The titre was calculated statistically with SAS version 9.4 using Wilcoxon rank sum test, for reasons that we barely collected the lowest serum titers during data collected when the titers of sample are lower than 1:4. The dataset contains ranked data, so rank sum test is helpful to evaluate the difference and data significance. A serum sample was considered positive if the neutralization antibody was observed at a dilution of 1:8, and then the GMT was calculated.

### Temperature sensitivity assay

The temperature sensitivities of the five Xinjiang EV-A90 strains were assayed on a monolayer of RD cells in 24-well plates. To observe the growth of EV-A90 at different temperatures, each virus was inoculated into 10 tubes; 5 of them were incubated at 36 °C, and the rest were incubated at 39.5 °C. After absorption at 36 °C or 39.5 °C for 1 h, the unabsorbed virus was removed, and maintenance medium (100 μL) was added to each well. The plates were incubated again at 36 °C or 39.5 °C, respectively, and harvested at 5 different time points post-infection (4, 8, 12, 24, and 48 h). The 50% cell culture infectious dose (CCID_50_) was calculated by the end-point dilution method on an RD cell monolayer in a 96-well plate at 36 °C. Virus isolates showing a greater than two-logarithm difference in titre at the two temperatures were considered to be temperature-sensitive. The series of viral titres were recorded, and the growth curves were drawn in Origin, version 9.

### Nucleotide sequence accession numbers

The full-length genome sequences of the five EV-A90 strains (HT-MYH12F, HT-HTH08F, HT-PSH40F, KS-KSH13F, and KS-SCH05F) described in this study were deposited in GenBank under accession numbers MG253032–MG253036, respectively.

## Electronic supplementary material

Supplementary figure 1
