# Supplementary material for: Antigenic characteristics and genomic analysis of novel EV-A90 enteroviruses isolated in Xinjiang, China
Source: Sci Rep. 2018 Jul 6;8:10247. doi: 10.1038/s41598-018-28469-9 (PMC6035207; doi:10.1038/s41598-018-28469-9)
Supplement: Supplementary file 1 — Supplementary figure 1 [file 41598_2018_28469_MOESM1_ESM.docx]

**Antigenic characteristics and genomic analysis of novel EV-A90 enteroviruses isolated in Xinjiang, China**

Keqiang Huang^1^, Yong Zhang^1^*, Yang Song^1^, Hui Cui^2^, Dongmei Yan^1^, Shuangli Zhu^1^, Qiang Sun^1^, Haishu Tang^2^, Dongyan Wang^1^, Wenbo Xu^1,3^

^1^WHO WPRO Regional Polio Reference Laboratory and Ministry of Health Key Laboratory for Medical Virology, National Institute for Viral Disease Control and Prevention, Chinese Center for Disease Control and Prevention, Beijing, People’s Republic of China

^2^Xinjiang Uygur Autonomous Region Center for Disease Control and Prevention, Urumqi City, Xinjiang Uygur Autonomous Region, People’s Republic of China

^3^Anhui University of Science and Technology, Anhui Province, People’s Republic of China

*Corresponding author: WHO WPRO Regional Polio Reference Laboratory and Ministry of Health Key Laboratory for Medical Virology, National Institute for Viral Disease Control and Prevention, Chinese Center for Disease Control and Prevention. Mailing address: No. 155, Changbai Road, Changping District, Beijing 102206, People’s Republic of China

E-mail: yongzhang75@sina.com


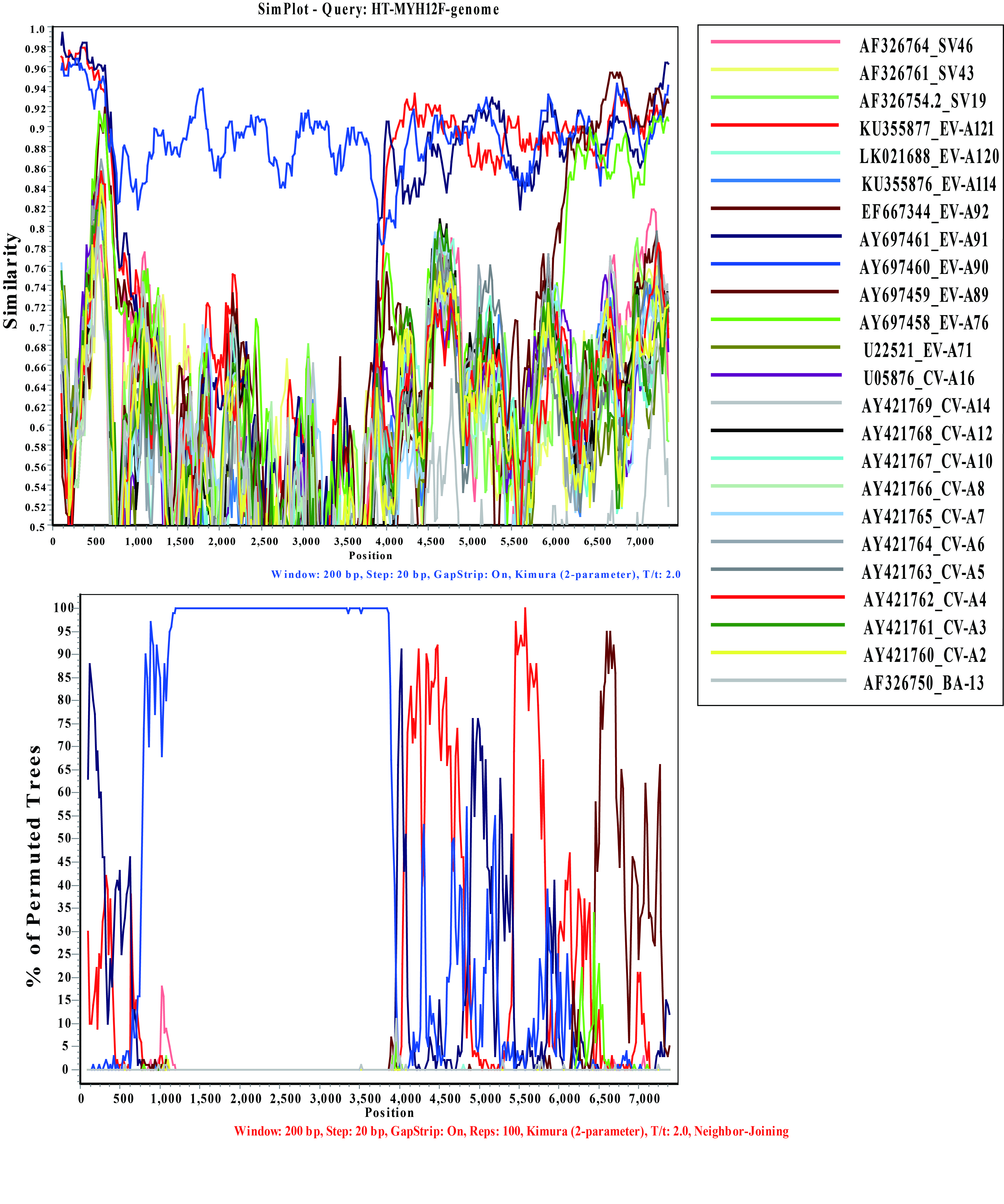

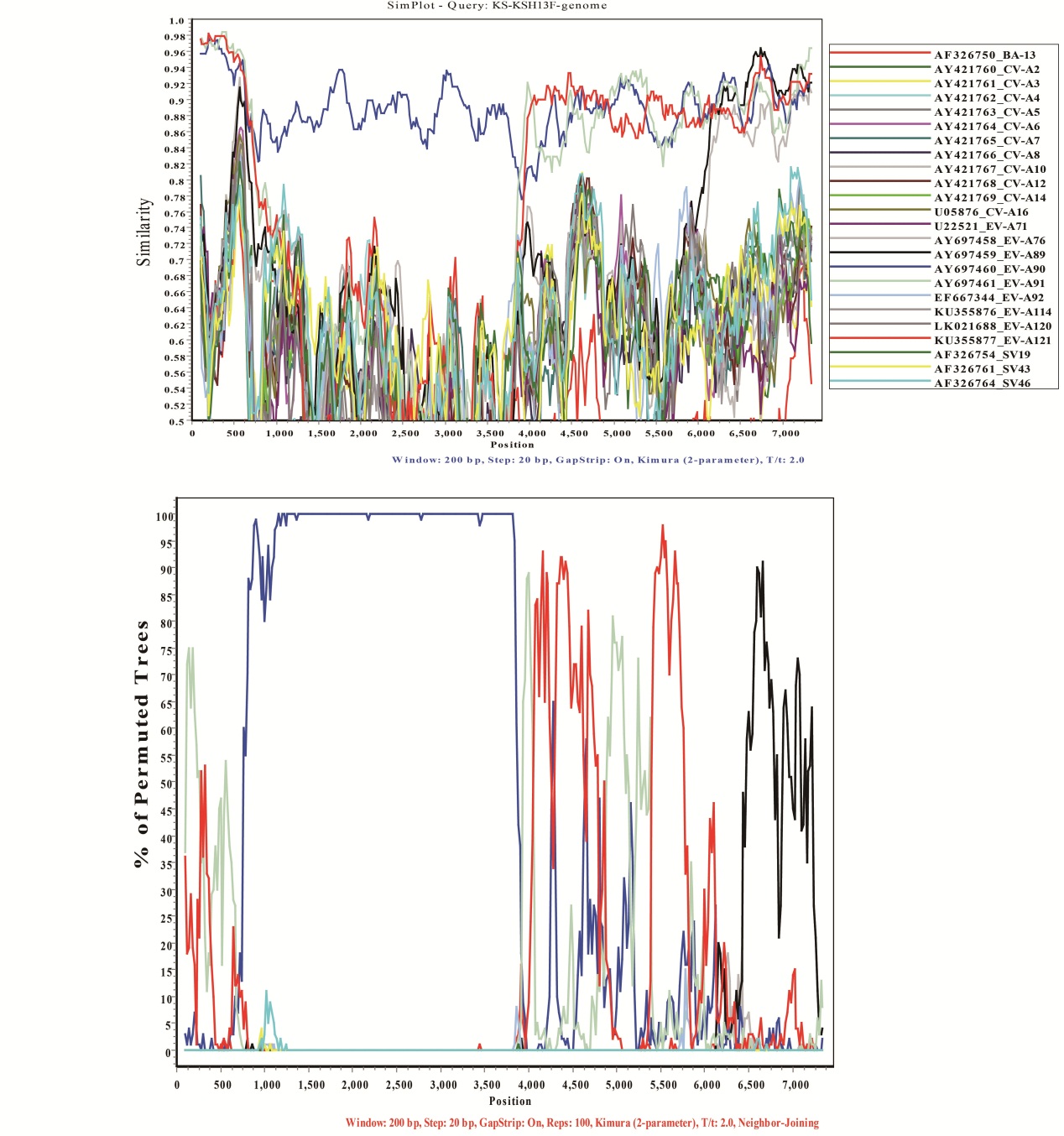

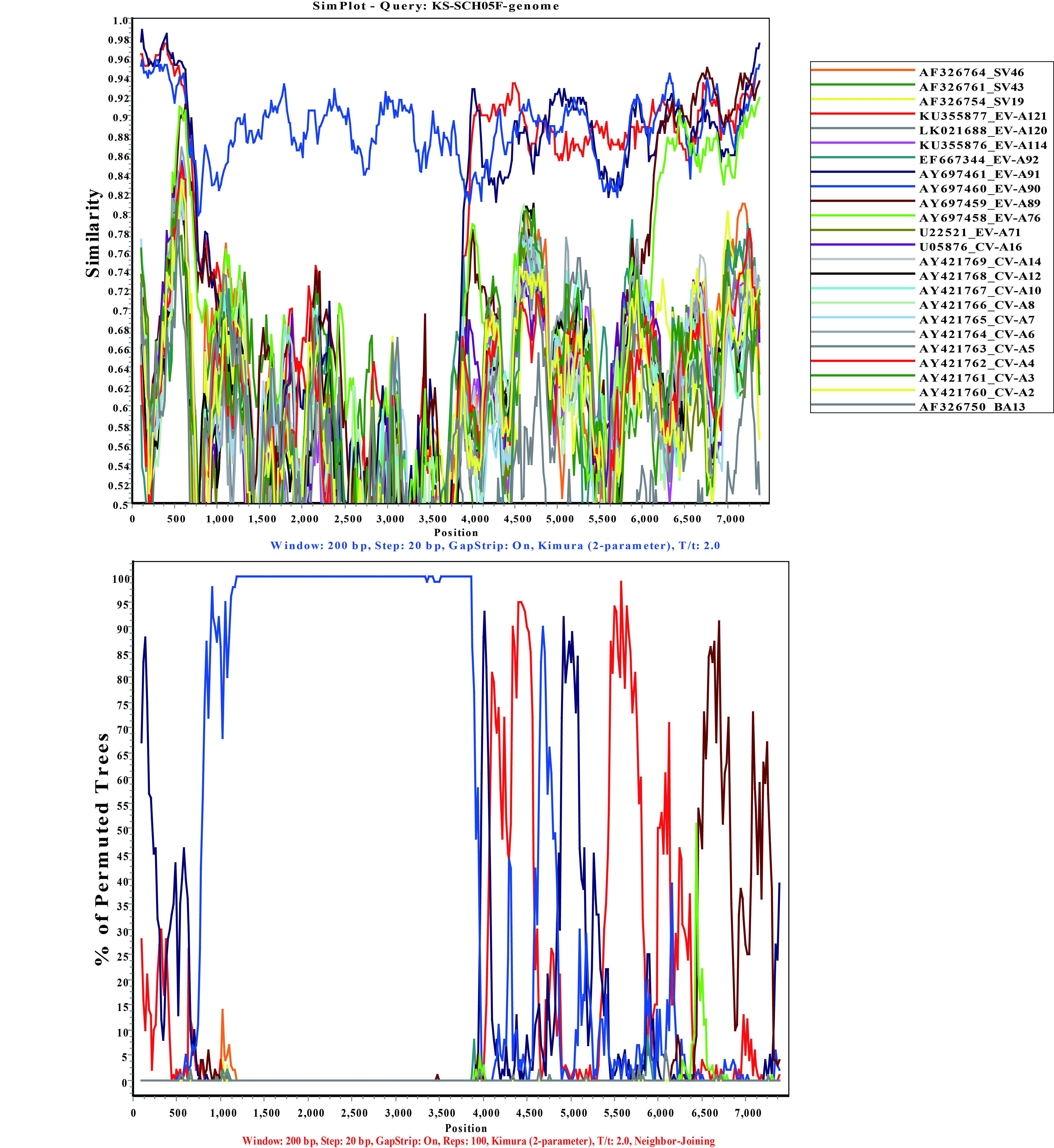

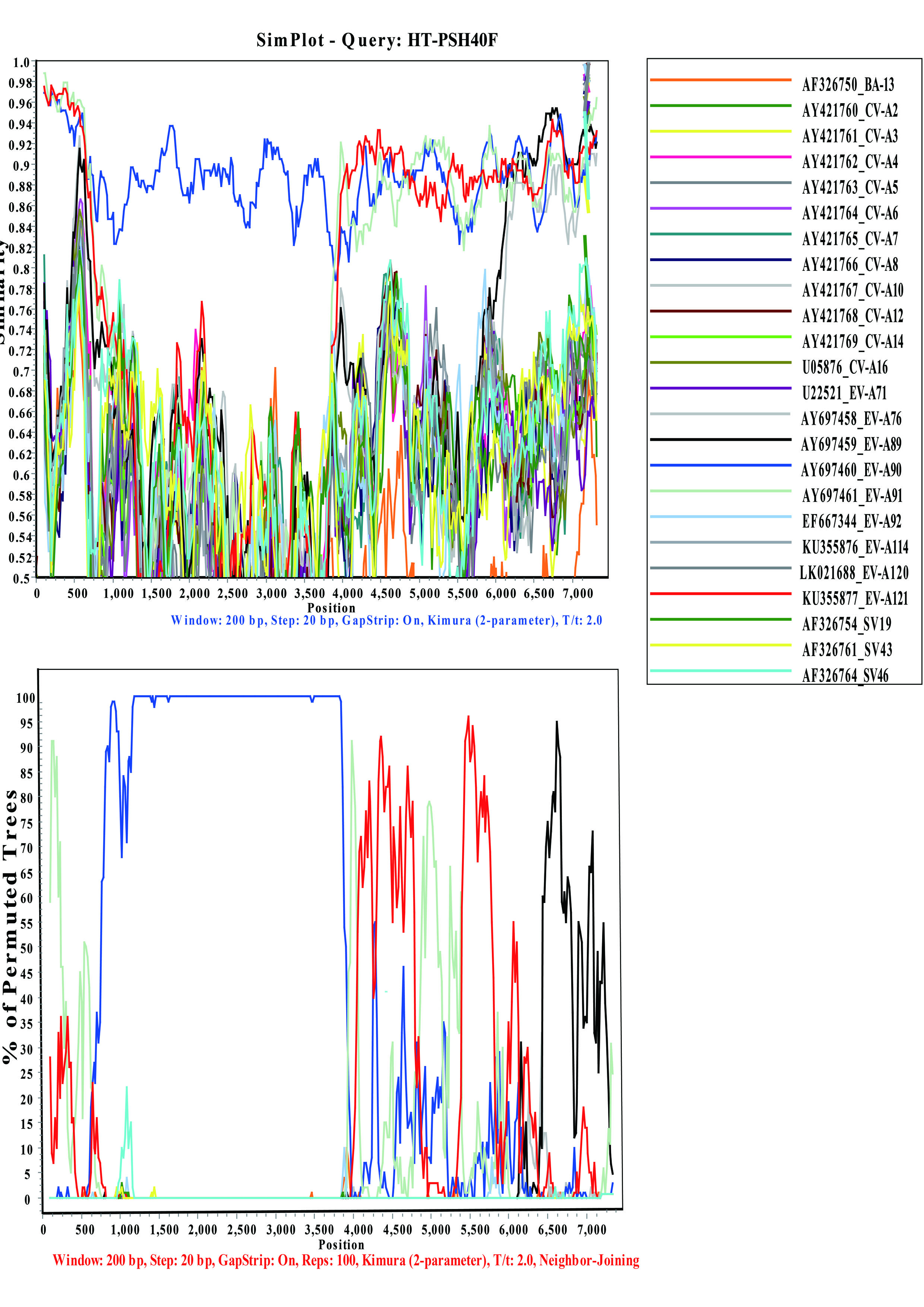


Supplementary Figure. Potential recombination analysis of the whole genome of other four Xinjiang EV-A90 strains. Similarity analysis (a) and bootscaning analysis (b) were performed in a 200-nt sliding window. Each point indicated similarity between the Xinjiang strain and other EV-A strains in a 20-nt moving step. Kimura (2-parameter) model was used in the analysis.
